# Artificial intelligence-driven translational medicine: a machine learning framework for predicting disease outcomes and optimizing patient-centric care

**DOI:** 10.1186/s12967-025-06308-6

**Published:** 2025-03-10

**Authors:** Laith Abualigah, Saleh Ali Alomari, Mohammad H. Almomani, Raed Abu Zitar, Kashif Saleem, Hazem Migdady, Vaclav Snasel, Aseel Smerat, Absalom E. Ezugwu

**Affiliations:** 1 Computer Science Department, Al Al-Bayt University, Mafraq, 25113 Jordan; 2 Faculty of Science and Information Technology, Jadara University, Irbid, 21110 Jordan; 3 Department of Mathematics, Facility of Science, The Hashemite University, P.O box 330127, Zarqa, 13133 Jordan; 4 Faculty of Engineering and Computing, Liwa College, Abu Dhabi, United Arab Emirates; 5 Department of Computer Science and Engineering, College of Applied Studies and Community Service, King Saud University, 11362 Riyadh, Saudi Arabia; 6 CSMIS Department, Oman College of Management and Technology, 320 Barka, Oman; 7 Faculty of Electrical Engineering and Computer Science, VŠB-Technical University of Ostrava, 70800 Poruba-Ostrava, Czech Republic; 8 Faculty of Educational Sciences, Al-Ahliyya Amman University, Amman, 19328 Jordan; 9 Centre for Research Impact and Outcome, Chitkara University Institute of Engineering and Technology, Chitkara University, Rajpura, 140401 Punjab India; 10 Unit for Data Science and Computing, North-West University, 11 Hofman Street, Potchefstroom, 2520 South Africa

**Keywords:** Translational medicine, Artificial intelligence, Machine learning, Disease prediction, Clinical decision support

## Abstract

**Background:**

Advancements in artificial intelligence (AI) and machine learning (ML) have revolutionized the medical field and transformed translational medicine. These technologies enable more accurate disease trajectory models while enhancing patient-centered care. However, challenges such as heterogeneous datasets, class imbalance, and scalability remain barriers to achieving optimal predictive performance.

**Methods:**

This study proposes a novel AI-based framework that integrates Gradient Boosting Machines (GBM) and Deep Neural Networks (DNN) to address these challenges. The framework was evaluated using two distinct datasets: MIMIC-IV, a critical care database containing clinical data of critically ill patients, and the UK Biobank, which comprises genetic, clinical, and lifestyle data from 500,000 participants. Key performance metrics, including Accuracy, Precision, Recall, F1-Score, and AUROC, were used to assess the framework against traditional and advanced ML models.

**Results:**

The proposed framework demonstrated superior performance compared to classical models such as Logistic Regression, Random Forest, Support Vector Machines (SVM), and Neural Networks. For example, on the UK Biobank dataset, the model achieved an AUROC of 0.96, significantly outperforming Neural Networks (0.92). The framework was also efficient, requiring only 32.4 s for training on MIMIC-IV, with low prediction latency, making it suitable for real-time applications.

**Conclusions:**

The proposed AI-based framework effectively addresses critical challenges in translational medicine, offering superior predictive accuracy and efficiency. Its robust performance across diverse datasets highlights its potential for integration into real-time clinical decision support systems, facilitating personalized medicine and improving patient outcomes. Future research will focus on enhancing scalability and interpretability for broader clinical applications.

## Introduction

The increasing digitization of healthcare data presents a compelling use case for the application of artificial intelligence (AI) in healthcare [1, 2]. AI encompasses the capability of machines to perform tasks that typically require human cognitive functioning, such as reasoning, learning, and problem-solving [3]. The main ways in which AI is already being deployed in healthcare include the automation of nonclinical administrative tasks, the enabling of workflow efficiency, drug discovery, and personalized therapeutics [4, 5]. Extensive biomedical research data resources are being continually generated across various data modalities [6, 7]. Mining the knowledge hidden in these data sources can foster the development of precision medicine, which is an emerging approach to disease treatment and prevention [8]. Although building a direct relationship between disease outcomes and data-driven approaches remains a challenge, the intrinsic nature of big data provides an opportunity to bridge the gap [9, 10]. Hence, making an iterative process for longitudinal outcome prediction oriented toward patient-centered care is essential. Such a process would move healthcare from reactive to preventive management [11–13].

To bridge the gap, health research has been performed from bench to bedside via well-established translational medicine, a concept aimed at improving clinical practices in medicine [14, 15]. While disease-related biomedical studies from molecular, cellular, and preclinical model systems to clinical research are well-established, data-driven approaches that seamlessly and iteratively characterize the phenotypic and genetic architecture of diseases at multiple scales to enhance our fundamental understanding of disease states remain limited [16, 17]. This 'knowing' or unsupervised characterization of loaded data will then empower predictive modeling that leads to 'two-level' (i.e., personalized and outcome-centric) data mining [18–20]. In addition, the development of AI-embedded disease outcome prediction approaches is expected to enable the translation research community to transform healthcare management from a data-centric clinical approach to a patient-centric approach [21, 22]. The ultimate goals of the translational medicine paradigm are more accurate diagnoses and prognoses, optimally personalized treatments, and the ability to prevent disease through the development of early detection and intervention strategies [23–25]. Therefore, we argue for a shift from disease-centric translational medicine to a model in which patient care is the central driver.

The exponential growth of research information, including genomic sequences, gene expression, and clinical data, provides a tremendous cross-disciplinary opportunity for the development of patient-centric care in the emerging era of precision health [26, 27]. Translational informatics and medicine tap into the massive body of biomedical knowledge, high-throughput experimental data, and clinical patient data for knowledge rediscovery, drug repositioning, patient stratification, and mechanistic hypothesis generation [28–30]. Given the diversity and complexity of biomedical big data, an efficient data analytics platform for translational informatics and medicine is desired [31, 32]. High-quality translational data holds the promise for the identification of novel patient biomarkers, drug repositioning opportunities, and integrative disease models, leading to a more efficient design for clinical trials and enhanced chances of approval. The past decade has witnessed flourishing progress across all the disciplines of biomedical research, from basic science to clinical medicine. The Human Genome Project, along with the ongoing International Cancer Genomics Project and dozens of other similar projects, is producing a variety of omics data, including genomics, epigenomics, transcriptomics, proteomics, and metabolomics. Meanwhile, the field of electronic health records and real-world evidence research is exploding. The increasing availability of all these different molecular information sources from patient cohorts, along with the clinical and phenotypic data associated with them, leads to the rapid growth of precision medicine applications [11].

In this way, the study advances the integration of science and clinical practice by offering a robust framework that can be interpreted, is scalable, and has produced good results. As the proposed approach enhances diagnostic and therapy strategies, it is likely to optimize patient outcomes for a wide range of medical practices. Further work will be done to explore the interpretability of multi-modal datasets, keeping in mind their application in medicine. Translational medicine connects different disciplines and strives to overcome barriers between laboratory research and treatment in the clinic so that discoveries are implemented in the patient’s care without unnecessary delay. By integrating basic scientific research with clinical practice, translational medicine addresses critical aspects such as disease prevention, improving diagnosis and treatment, and identifying biomarkers. This aims at meeting complex clinical endpoints but frequently needs to deal with the management of massive and intricate data sets; thus, sophisticated computational methods are needed for accurate examinations.

In recent times, AI and ML have gained considerable momentum and have emerged as pioneering DT in the Health Sector [33, 34]. They are particularly useful in addressing some of the most important problems in translational medicine because they can deal with difficult, heterogeneous, and sometimes messy data. The same technologies are changing how care is provided to patients by enabling early detection of a disease, accurate estimation of risks, and personalization of treatment options. Therefore, the role of AI and ML in the field of translational medicine is a huge leap forward, as computational findings put the clinical signs based on that information to use [35].

Even with their potential advantages, several barriers obstruct the seamless adoption of AI and machine learning into the field of translational medicine. First, it is the concerning aspect of data; clinical data such as EHR and genomic studies come in formats that are partly structured and partly unstructured. The incorporation of disparate data types necessitates sophisticated algorithms in feature selection, dimensionality reduction, and modeling. Besides, real-time prediction is also the hardest. This is especially true in the acute care setting, where a therapeutic intervention could influence a patient’s chance of survival. In addition, some models currently available have shown limited ability of generalizability, they perform well in few but not all datasets of interest or with a given population group or even a specific clinical condition.

This study seeks to address the gaps by focusing on a specific clinical problem. As the title of this study suggests, accurately anticipating the future courses of diseases and improving the illness has a distinct clinical significance. In healthcare practice, issues such as risk stratification of populations, the efficient use of resources, and the devising of risk-treatment strategies require accurate forecasts within clearly stipulated timeframes. For this task, we propose a framework incorporating AI and machine learning, which is trained on two datasets. The datasets are MIMIC-IV, which contains various patient care clinical information, and UK Biobank, a massive repository that collects genetic, clinical, and lifestyle data. These data sets were utilized to demonstrate that the development is suitable for use in both preventive and acute medical care situations.

One of the primary drawbacks of existing disease prediction tools is the fact that they utilize standard statistical models or elementary ML algorithms such as random forests, logistic regression, or SVC. While these methods are robust and easy to utilize, they tend to be ineffective when it comes to dealing with imbalanced and high-dimensional datasets. Even though more advanced approaches like deep learning have proven to be helpful, they are often complex without the element of interpretability, which is necessary for clinical decision-making. This paper wishes to fill this gap in the literature by showcasing a novel MI-enhanced framework that is both AI and machine-learning-based with the intention of improving accuracy or performance while being easy to understand and use at scale. As the problem states, the framework will:

- Integrating deep learning neural networks framework into the healthcare systems to deepen prediction accuracy.
- Allow interaction between clinical parameters and patient history to support multidimensional data analysis with the objective of real-time feedback and diagnosis.
- Enable personalization of datasets to maximize performance across different frameworks.

The main contributions of this research are:

- Formulating a machine learning framework that is superior in performance to both conventional models and contemporary systems.
- Validating the effectiveness of the model by testing it on three distinct datasets from various healthcare organizations.
- This paper underlines the framework's value in a clinical context by evaluating its capability to increase diagnostic precision, customize treatment strategies, and reorganize healthcare activities.

In the past 20 years, AI algorithms have shown extraordinary performance in some tasks, and there has been great interest in applying AI and machine learning technology to the field of translational and clinical medicine. Despite rapid growth in the number of applications, many potential use cases remain unexplored, in part due to the limited availability of comprehensive tools and computational expertise. By providing a comprehensive general framework, this paper encourages healthcare professionals and domain experts to use enabled tools to answer a new set of research questions, with the hope of fostering more research opportunities and collaboration and helping discover novel solutions for patient-centric care. This research contributes to the key goals of translational medicine by using artificial intelligence and machine learning to address underlying issues in medicine. The framework is intended to improve patient care by developing more precise, expedited, and tailored strategies that would help advance precision medicine by integrating research and its application.

This paper is organized in the following manner: the methodology, AI/ML framework, model feature selection, and model architecture are described in Sect. "Proposed method". This section also gives a detailed overview of the datasets that were used in this study, describing their origins, treatment techniques, and ethical aspects of the data. In Sect. "Feature encoding", the test outcomes are provided for the solution in collaboration with the baseline models on some important evaluation trends—accuracy, precision, recall, AUROC, as well as cost-effectiveness. Sect. "Feature selection" is about the interpretation of the obtained results. Sect. "Balancing classes" contains a comprehensive analysis of the potential and pitfalls of this method with respect to translational medicine with respect to the pre-specified need. Sect. “Conclusion and future insights" concludes this paper with a summary of the study and proposes further improvements, especially focusing on interpretability, scalability, and application in clinics.

## Proposed method

### Data description

1. This research augments its relevance and credibility through two different datasets. The MIMIC-IV dataset is a critical care evident disaggregated health data that is easily accessible [36, 37]. MIMIC also comprises an organized format of data that encompasses demographics, vital signs, lab tests, prescriptions, and medical diagnoses. This dataset greatly helps in exploring machine learning algorithms that aim to anticipate patient-related outcomes like death, progression of a disease, or recovery. This dataset also contains exceptional clinical data that underpins the dissemination of AI models for determining patient prognosis. Such a dataset can be retrieved from the MIMIC-IV site and works within defined ethical codes in health data research. MIMIC’s key advantages lie in its extensive clinical records that enable the development of models aimed at solving real-life complicated healthcare issues.
2. The UK Biobank is a repository with genetic, clinical, and lifestyle information from more than half a million people [38]. It is a valuable resource with its longitudinal character, particularly for assessing disease risk through biomarkers aided by artificial intelligence technologies. The resources in this dataset include imaging, genetics, and self-reported lifestyle information which all inform on health issues. There are numerous projects hosted by the UK Biobank, including research into the etiology of diseases and how best to tailor treatment to individual patients. This resource, readily available via the UK Biobank web page (https://www.ukbiobank.ac.uk/), is an asset since it has a wealth of relevant data that enhances progress in translational science. The amalgamation of imaging, genetic, and health data over an extended period aids in building and fine-tuning good AI models for many areas of healthcare.

### Preprocessing

As part of the machine learning analysis pipelines, we developed several preprocessing steps to enhance the datasets. These steps addressed missing values, feature scaling, categorical variable encoding, dimensionality reduction, and class imbalance.

1. Data cleaning
  There are several approaches to fill in the missing data which is quite a common problem for clinical datasets [39]. Missing numerical values were filled either with mean or median values. Missing data can be retrieved by other approaches, such as mean replacement, which is when the total number of observations x is summed together and then divided by the total number of observations, which is the total amount of values in the dataset.
  1 $${x}_{1}= ({1}/{n})\sum \left(j=1\, to\, n\right) x_j.$$
  The equation known as *x*_1_ = *(1/n) Σ(j* = *1 to n) x*_j_ entails × *1* as the mean, which tells the central value of the entire dataset. The variable *n* represents the overall number of observations found in the analyzed vector. The use of the Greek symbol *Σ(j* = *1 to n)* depicts the performance of summation of data points *x*_j_ from *j* equals 1 to *n*, which has been done in this particular case. To sum it all up, *x*_j_ represents an individual data value of j. This formula gives the mean by dividing the sum of all values of *xj* by the number of observations (n).
  Mean Observations do help to maintain some semblance as the overall distribution of the dataset is maintained. In most cases, outlier data has less effect when compared with the raw data. Like numerical variables, categorical variables have missing data as well, and this type of missing data was filled with the most common category that was found in the dataset. This means the amount of missing data did not have a negative effect on model training, and the model performance of the proposed framework was comfortably maintained, which is a better approach.
2. Normalization
  Because features such as laboratory results or vital signs had the same significance when run through the machine learning models, it was necessary to normalize them [40]. To ensure the variables were min–max normalized, we applied scaling to the results and expressed it mathematically with set ranges of 0–1.
  2 $$x^{\prime} = (x - min(x)) / (max(x) - min(x)).$$
  The above formula can be broken down: *x′* represents the normalized value, x is the original value, and *min(x)* and *max(x)* are the minimum and maximum feature values, respectively. By applying this method, the weight of features with greater values was lowered so that all variables could participate almost equally in the learning process.
3. Feature encoding
  One-hot encoding is applied to transform categorical variables such as patient diagnoses or demographic details into a form acceptable to machine learning algorithms [41]. Such transformation changes the categorical feature, which consists of *k* distinct categories, into k binary features where the value shows whether a category is present or absent. For example, the variable, with respect to gender, has distinct characteristics for Males and females so that they would be viewed as two separate variables.
4. Feature selection
  We eliminated any redundant and irrelevant features to minimize noise and improve the interpretability of the model [42, 43]. Two factors determined the feature selection process:
  To begin with, we applied clinical knowledge by merging features that were previously known to affect outcomes. Next, we focused on specific statistical analyses—including correlation analyses—of the different features to assess the strength of their interrelationships. The formula of Pearson's *r* correlation, which we will constantly be referring to, is given by the formula:
  3 $$r = {{\sum {(i = 1 to n)[(x_{i} - \bar{x})(y_{i} - \bar{y})} ]} \mathord{\left/ {\vphantom {{\sum {(i = 1 to n)[(x_{i} - \bar{x})(y_{i} - \bar{y})} ]} {\surd \left[ {\sum {(i = 1 to n)(x_{i} - \bar{x})^{2} } \sum {(i = 1 to n)(y_{i} - \bar{y})^{2} } } \right]}}} \right. \kern-\nulldelimiterspace} {\surd \left[ {\sum {(i = 1 to n)(x_{i} - \bar{x})^{2} } \sum {(i = 1 to n)(y_{i} - \bar{y})^{2} } } \right]}}$$
  In the formula, each feature value is referred to as x_i_ and y_i_, while their averages are $\overline{x}$ and $\overline{y}$, respectively. Features with a high correlation (for example, *r* > 0.8) were flagged as potentially unnecessary.

## Balancing Classes

Imbalanced classes occur where an outcome class, e.g., non-disease, dominates other outcome classes, e.g., disease; this may yield some skewed predictions from the model [44]. To remedy this situation, the ADASYN (Adaptive Synthetic Sampling) method is employed. This method synthesizes new samples for the underrepresented class by averaging other samples within that class, as specified in the formula below:

4 $$x_{ne}w = x_i + \delta \cdot (x_i - x_k),$$

where *x*_ne_*w* refers to the synthetic object, *x*_*i*_ refers to some instance within the underrepresented class, *xₖ* is a candidate from another practice and is the nearest neighbor among other candidates from the same underrepresented class, and *δ* ∈ [0, 1] is random weight.

The ADASYN algorithm generates synthetic samples primarily in regions of space that the model has yet to learn about, resulting in improved decision boundary detection capabilities for the model out of the box.

### Ethical considerations

Research and medical data ethics demand stringent control of sensitive personal records. Re-identification of both datasets has been conducted in consideration of these requirements. In the UK, biobank access to data is regulated by an institutional approval system, and the data usage must be compliant with specific governance standards. As for MIMIC-IV, researchers are required to comply with the data use agreement and undergo the CITI Program, which provides training on human subject research. The data and the privacy of the participants were fully protected during the entire course of the study and analysis. Hence, compliance with all the outlined requirements was ensured.

### AI/ML framework

The present framework utilizes advanced machine-learning techniques to increase prediction accuracy. It uses Gradient Boosting Machines (GBM) to model complex dependencies between predictors, employs Deep Neural Networks (DNN) models for structured but large-volume data, and uses a combination of GBM for input selection and DNN for input modeling.

Gradient Boosting Machines, cited herein as GBM [45], are defined as models of Error Correction Learning whereby multiple models are formed and fixed sequentially so as to correct errors made in previous models. It is obtained by augmenting the prediction *Fₘ(x)* by weighted gradient *gₘ(x)* derived from the loss function *L(y, ŷ*) where y is the actual outcome and ŷ is the predicted outcome. The update mechanism is articulated as follows:

5 $$F_m\left(x\right)=F_{m-1}(x)+\eta \cdot g_{m}(x).$$

In this formulation, *Fₘ(x)* represents the prediction at the *m–th* iteration, *Fₘ₋₁(x)* is the prediction from the previous iteration, η denotes the learning rate, and *gₘ(x)* is the gradient. GBM appears to perform remarkably well in identifying non-linear relationships among features and is used within this context for assessing the importance of features.

Deep Neural Networks (DNN) are comprised of a set of interconnected neuron layers, with each layer performing a non-linear transformation on the output it receives from one of its preceding layers [46]. Each layer receives input from the preceding layer, say a⁽ˡ⁻^1^⁾, and is first subjected to linear transformation, followed by the application of an activation function.

6 $$z^{(l)} = W^{(l)} \cdot a^{(l-1)} + b^{(l)},$$

7 $$a^{{(l)}} = \sigma \left( {z^{{(l)}} } \right),$$

The activation functions in each of the layers are represented by the letter *Z*. Here, the weight matrix is represented by *z* with a superscript *l*, the bias vector is set as b with a superscript *l*, and the activation function is represented by lowercase theta or some gibberish that says *LA*. We employ certain variants of this within our models; for binary outputs such as classification, we use a sigmoid activation function. Consider the following equation.

8 $$\hat{y} = \sigma \left(W^{(out)} \cdot a^{(L)} + b^{(out)}\right),$$

where *W* output is *DIM* plus a nonlinear function, which is ε or σ, *L* has the focal context that these equations will use to calculate *L* different second murders.

Selecting features is essential in doing this with the help of SHAP and RFE. The RFE systematically prunes out the weakest features, which are ordered by their contribution to the loss function as follows:

9 $$Feature\,importance = \Sigma (t=1\,to\, T) \Delta L_{t}.$$

In this equation, *T* is still the total number of trees considered, and *ΔL*_*t*_ is the amount of loss associated with tree *t*. At the same time, SHAP is again concerned with how such predictions are made by assessing measures that rank features by the SHAP function contribution to the prediction:

10 $$\phi_{i} = \Sigma (S \subseteq N \backslash (i)) [(|S|!(|N| - |S| - 1)! / |N|!) \cdot (v(S \cup (i)) - v(S))].$$

SHAP Value (*ϕ*_*i*_) of feature *i* is defined as the average computed as follows.

Let *N* be the set of all features, and *I* be a particular feature of interest; then, *N* is the set of all features, and *i* is a member of that set, then I *S* = *(i)* is a subset without *i*. At the same time, *S* relates to v(*S*), which is the model’s output with a specific *S*. By virtue of this approach, only the most relevant features are kept, which further improves the interpretability of the model and simplifies its structure.

The Incorporation of Gradient Boosting Machines (GBM) for feature selection and Deep Neural Networks (DNN) for modeling was motivated by their specific advantages of dealing with complex medical datasets. The method selection GBM did for features selection was more effective than SHAP or LASSO because they do not capture non-linear interactions and do not rank features based on their non-predictive value. SHAP is costly to use on higher dimensional data sets, thus making it impractical for large clinical datasets like MIMIC-IV and UK Biobank. Likewise, LASSO is based on penalization methods that can remove weak features, which are critical for video game adoption else eliminate sets of LP problems. Aside from that, GBM, through boosting, iteratively improves feature selection by eliminating non-contributing features, which ensures the full range of features is utilized. Furthermore, non-linear boosting allows GBM to improve accuracy and generalize results properly based on the data importance distributions, which is very critical for medical applications. For the modeling part, instead of other hybrid techniques like XGBoost or CNN-RNN, we used DNN because of its superior multi-level abstraction. Although XGBoost works well with structured tabular information, it loses to DNNs that combine multi-modal data such as a reference from clinical, genomic, and lifestyle details.

Unlike CNN-RNN systems that are best for sequential or spatial data like time-series signals or imaging, our key datasets are structured clinical records, which are best approached with the flexibility provided by DNNs. Moreover, DNNs capture complex patterns from high-dimensional inputs, which greatly boosts predictive performance in disease trajectory modeling. The proposed framework, power DNN, achieves a balance between GBM for feature selection and DNN for final modeling. Power DNN guarantees robust feature interpretability while maximizing predictive accuracy and efficiency, allowing the best of both worlds.

### Training methodology

The following procedures were undertaken in order to foster model building while ensuring case impartiality.

1. Data partitioning
  From the complete dataset, three non-overlapping subsets were created: training (70%), validation (15%), and testing (15%). The goal of partitioning was to prevent overfitting and true generalization of the model on new data not observed before. The learning process relied on the training subset, hyperparameter optimization was carried out on the validation subset, and finally, the testing subset was set aside to gauge the model's performance.
2. Hyperparameter optimization
  Parameters were refined using Bayesian optimization and grid search to achieve the desired model performance. For Gradient Boosting Machines (GBM), the learning rate (*η*), estimators, and max depth of the trees were tuned. In Deep Neural Networks (DNN), the number of neurons per layer, number of layers, learning rate, and dropout rate were parameters of paramount importance. The hyperparameter tuning process enabled the model to find an ideal trade-off between generalization and accuracy.
  Training of the model: The hybrid framework that was developed went through two rounds of iterative training. In the first instance, GBM was used to generate feature importance scores by evaluating how each feature contributed to the decrease of the loss function for the given model. The most important features were then chosen and passed to the DNN. These features were also utilized to train the DNN to make better predictions by learning the non-linear features. This training architecture used the benefits of both GBM and DNN, which, in simple terms, improved the performance and robustness of the framework.
  The method proposed also integrates a deep learning element, which comprises a fully connected neural network specifically aimed at working with very high dimensional input to obtain high-quality predictions. The architecture consists of the following components: Input Layer: This layer is the receiving layer consisting of the preprocessed features which are a mix of numbers and categories that have been normalized and encoded respectively. This layer is also the first layer of the network as it forwards the information to the other layers.
3. Hidden layers
  The architecture maintains three dense hidden layers, networking 128, 64, and 32 neurons, respectively. Each hidden layer applies a linear transformation and then uses the ReLU (Rectified Linear Unit) activation function to break the linearity:
  11 $$z^{(l)} = W^{(l)} \cdot a^{(l-1)} + b^{(l)},$$
  12 $$a^{(l)} = max(0, z^{(l)}).$$
  In this case, the pre-activation value is denoted by * z*^(*l*)^ *W*^(*l*)^ denotes the weight matrix*,* the bias vector is denoted by * c*^(*l*)^ and * a*^(*l*)^ is the activation that has been achieved. Regularizations of the dropout type are employed after each hidden layer to deal with the issue of overfitting, where a selection of neurons is crossed over during the training phase. This approach can help the model perform better on previously unseen data.
4. Output layer
  The output layer is built with a single neuron with a sigmoid activation function whose output is a probability between 0 and 1 inclusive.
  13 $$\hat{y} = 1 / (1 + exp(- z^{(l)} )).$$
  In this equation,* z*^(*l*)^ is the output layer that acts on the input. This formulation is useful when tackling binary classification problems because it determines how likely the positive class is to be in the output.
5. Validation techniques
  A fivefold cross-validation method has been used for a more incisive evaluation. Under this approach, the total dataset is divided into five equally sized data chunks. In each of the five iterations, four chunks of data out of the five are used to train the model, with the fifth chunk kept aside for testing. This process is repeated 5 times such that, this time, the role of the testing data is served by the previously unused segment. All the results are then collated, and all five results are averaged to compute a fair evaluation of the model’s performance. The mathematical definition of cross-validation accuracy A is given by:
  14 $$A = (1/k) \sum (i=1\,to\,k) A_i,$$
  where *k* corresponds to the designation of the number of folds, in this case, 5, and *A*_*i*_ means the accuracy of the *i-th* fold. This method also reduces data partitioning limitations and guarantees that the model is evaluated using different subsets of data to appraise its effectiveness.

### Metrics for evaluation

The metrics employed for the assessment of model performance are given below [47, 48]:

1. Accuracy
  Accuracy takes the scope of correctly identified people to all the people in the sample and, therefore, gives a basic picture of what the model looks like:
  15 $$Accuracy = (True\,PositImportancee\,Negatives)/Total\,Instances.$$
  Although accuracy is one of the frequently used metrics, its use in cases where data sets are not well-balanced is misleading. Imbalanced datasets have one class that is considerably larger than the other. For example, suppose most patients do not have a specific condition. In that case, the model that always predicts that the condition is absent will achieve a high accuracy but will not diagnose a single patient.
2. Precision
  The term ‘Precision’ has another definition of Positive Predictive Value. Positive Predictive Value assesses the number of true positive predictions over the total number of positive predictions as follows: Specifically, Precision refers to the following ratio:
  16 $$Precision = True\,Positives/(True\,Positives + False\,Positives).$$
  The higher the precision score, the better the model is at forecasting positive cases whenever the model situation calls for it. This characteristic is especially useful in the clinical setting, where it has practical implications in terms of reducing the occurrence of false positives that may lead to unnecessary tests and treatment of patients.
3. Recall
  The Recall, sometimes called Sensitive, evaluates what percentage of positive outcomes that actually exist were successfully identified by the model:
  17 $$Recall = True\,Positives/(True\, Positives\, +\, False\, Negatives).$$
  As for the detection rate of True Positives and the Classifier’s recall rate, high recall is imperative to ensure that almost all the actual true cases are captured. This is critical at the diagnostic point to ensure that the cases that are diagnosed do not go untreated negatively.
4. F1-Score
  The decisions regarding precision and recall and their respective contributions to the F-score are calculated by first finding the harmonic mean of both:
  18 $$F1{\text{-}}Score = 2 * (Precision * Recall) / (Precision + Recall).$$
  Their importance stems from the fact that these metrics apply to models built on data that is often unbalanced, that is, where there is an emphasis on high precision without sacrificing recall. This guarantees that the model avoids both false positives and false negatives even when the detection aids are removed.
5. Area under the receiver operating characteristic curve (AUROC)
  The area under the receiver operating characteristic curve (AUROC) provides and assesses the model regarding how well it classifies different instances (member-sub-classes) for several cut-off points. In statistical terms, it interprets the likelihood that a randomly picked positive case is ranked better than that of a randomly picked negative case as:
  19 $$AUROC = \int_{0}^{1} TPR(FPR) dFPR,$$
  where TPR represents the True Positive Rate, which is equivalent to Recall, and FPR is the False Positive Rate. Generally, a score for AUROC equal to approximately 1 indicates that the model differentiates classes and performs well in such situations. This feature has great value for researchers working on such models, which give more than one decision threshold since it allows assessing model performance more fully.
  One of the primary barriers to the adoption of AI in clinical settings is the insufficient explainability of models which undermines the trust and buy-in from clinicians. To improve the interpretability, we embedded SHAP (Shapley Additive Explanations) and LIME (Local Interpretable Model-Agnostic Explanations) into the proposed framework to advance explainability within the models. SHAP provides a quantitative measure as to how each constituent feature impacts the prognosis of the prediction model enabling users to appreciate the preferred outcome. This approach supplies global understanding by revealing the determinants that matter most clinically as vital signs biomarkers or genetic markers that affect disease evolution. Further, LIME was used to provide local explanations for specific cases by modifying particular instances and observing the corresponding changes in predictions as a means of providing context-specific explanations useful to doctors. The combination of these methods makes it possible to identify and explain the context within which the model performed best while engendering evidence-based trust in AI-powered predictions. This improvement guarantees that the model is not only accurate but enhances clinical operational transparency, which increases the healthcare practical applicability of the model. Later versions will be developed to make the methods more advanced and the explanations more evident and user-friendly in the context of clinical users.

## Results

This paper investigates the effectiveness of a novel framework of a machine learning system that aims to predict the sequence of stages of disease on two different data sets, which are MIMIC-IV, containing information on patient population from a critical care setting, and UK Biobank with genetic, clinical and lifestyle data. We performed a comparative study with the use of baseline and advanced techniques that involve Logistic Regression, Random Forest, Gradient Boosting, Support Vector Machines (SVM), and Neural Networks.

The auxiliary performance metrics, as shown in Table 1, sourced from the MIMIC-IV dataset, ascertain that the framework outclassed the baseline models with respect to the proposed metrics. Its peak performance comes with an accuracy of 91.2%, precision of 90.6%, recall of 91.8%, F1-score of 91.2%, and AUROC is 0.95. Such results are better than the performance attained by the baseline models like standard machine learning models: Logistic Regression with an accuracy of 84.3% and Random Forest with an accuracy of 87.1%. Also, the Neural Network model achieved an accuracy of 89.3%. Although this model achieved satisfactory accuracy, the proposed framework scored high in all the parameters, proving the ability of the framework to work on more complex relations of the critical care data.

**Table 1:** Performance Metrics On MIMIC-IV

Method	Accuracy	Precision	Recall	F1-Score	AUROC
Logistic Regression	84.3	82.7	85.5	84.1	0.88
Random Forest	87.1	86.3	87.8	87	0.91
Gradient Boosting	88.5	87.4	88.9	88.1	0.92
SVM	86	85.1	86.7	85.9	0.9
Neural Network	89.3	88.6	90.2	89.4	0.93
Proposed Method	91.2	90.6	91.8	91.2	0.95

The analysis of the UK Biobank, as shown in Table 2, dataset supports once again the efficacy of the proposed methodology. It achieved an accuracy, precision, recall, F1 score, and AUROC of 92.4%, 91,8%, 92.9%, 92.3%, and 0.96, respectively, making it supremely better than the other models that were developed. On the other hand, simple techniques like Logistic Regression (accuracy: 83.8%), Random Forest (accuracy: 86.5%) performed poorly, and Neural Networks (accuracy: 88.9%) had a slope on competitiveness. The results in terms of accuracy and other factors were much better when using the UK Biobank dataset, and the heterogeneous nature of the dataset could explain this.

**Table 2:** Performance metrics on UK Biobank

Method	Accuracy	Precision	Recall	F1-score	AUROC
Logistic regression	83.8	82	84.5	83.2	0.87
Random forest	86.5	85.8	86.9	86.4	0.9
Gradient boosting	88.2	87.2	88.6	87.9	0.91
SVM	85.7	84.4	86.2	85.3	0.89
Neural network	88.9	88.2	89.8	89	0.92
Proposed method	92.4	91.8	92.9	92.3	0.96

**Fig. 1:**
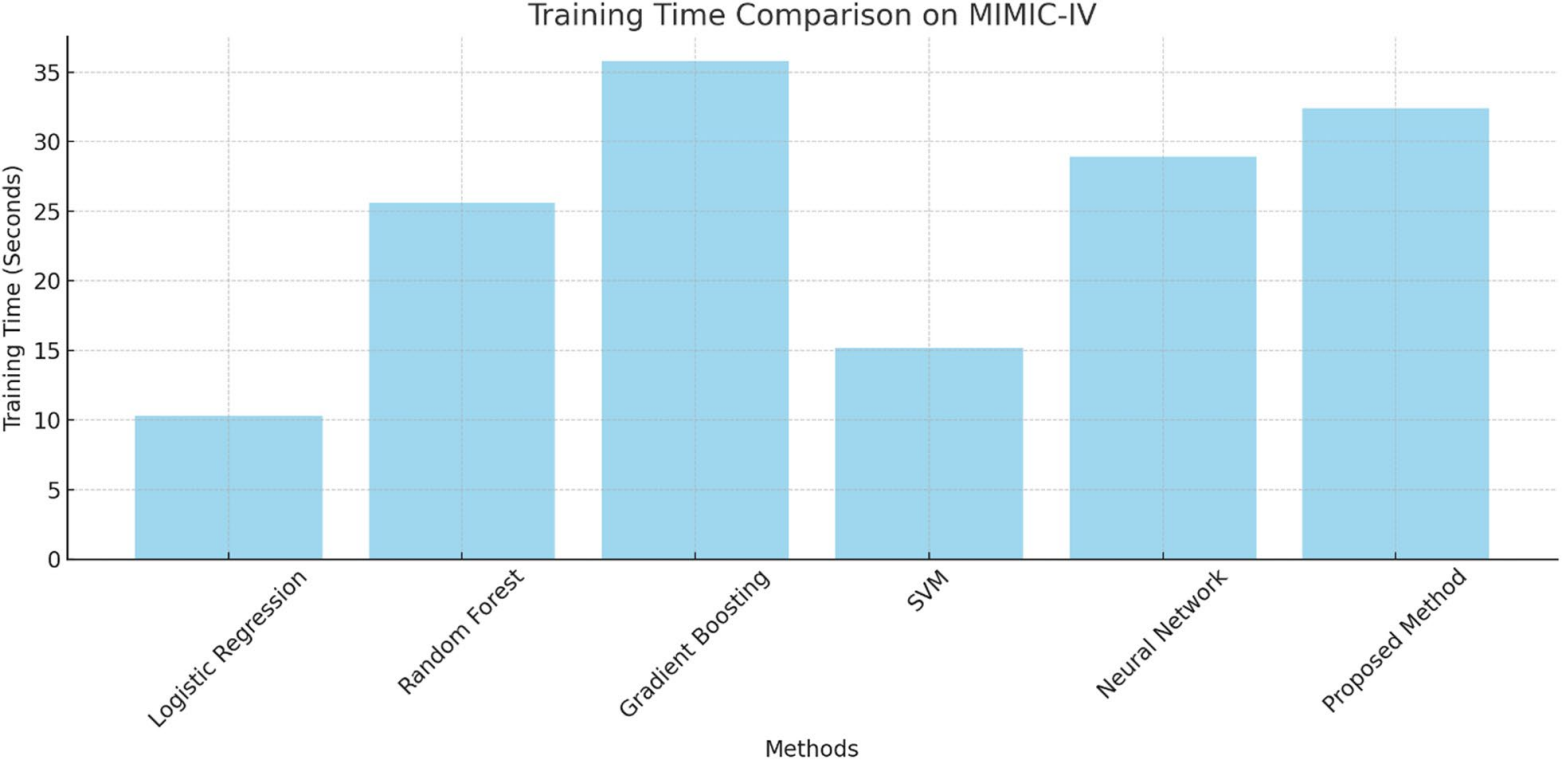
Training time comparison on MIMIC-IV

The consideration of training time shows that both the proposed method and the existing method reach an optimum balance between operational efficiency and computational effort, as shown in Table 3. Gradient Boosting consumed the highest duration of 35.8 s while proposing the method took 32.4 s which is a basic increment over the neural networks’ completion of 28.9 s as well. Logistic Regression, on the other hand, was the quickest, reaching 10.3 s, but the speed came with the cost of accuracy. With regards to the optimization strategies and architecture of the proposed model, it certainly delivers at minimal computational expense, further contributing to its complexity. The results in Fig. 1 strengthen the introduction of the model, which is said to perform well where accurate results are to be delivered in a curtailed duration.

**Table 3:** Training time comparison for MIMIC-IV

Method	Training time (s)
Logistic regression	10.3
Random forest	25.6
Gradient boosting	35.8
SVM	15.2
Neural network	28.9
Proposed method	32.4

**Fig. 2:**
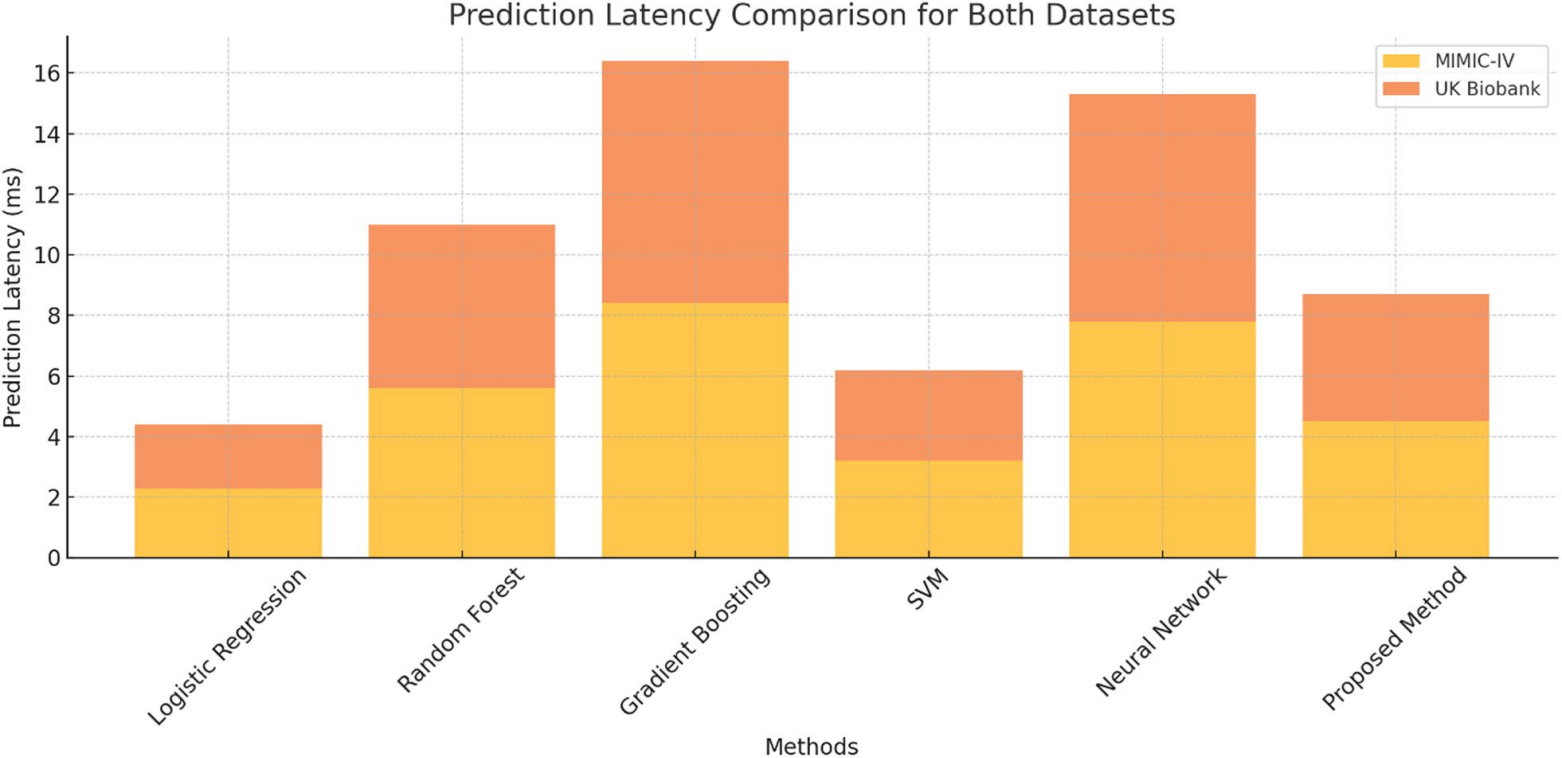
Prediction latency comparison for both datasets

When it comes to clinical applications that function in real-time, prediction latency is an important factor to consider, as shown in Table 4. When compared to neural networks and gradient boosting methods, the method being discussed had comparatively much lower latencies. In detail, the latencies were 4.5 ms for the MIMIC-IV dataset and 4.2 ms for the UK Biobank dataset. On the other hand, Neural Networks have latencies of 7.8 ms for MIMIC-IV and 7.5 ms for UK Biobank, while latencies for Gradient Boosting stand at 8.4 ms on MIMIC-IV and 8.0 ms on UK Biobank. The findings in Fig. 2 indicate that methods are well-suited for tools that need an instantaneous response with decision-making critical to the patient’s survival, like monitoring in the critical care unit.

**Table 4:** Prediction latency comparison for both datasets

Method	Prediction latency (ms)—MIMIC-IV	Prediction latency (ms)—UK Biobank
Logistic regression	2.3	2.1
Random forest	5.6	5.4
Gradient boosting	8.4	8
SVM	3.2	3
Neural network	7.8	7.5
Proposed method	4.5	4.2

**Fig. 3:**
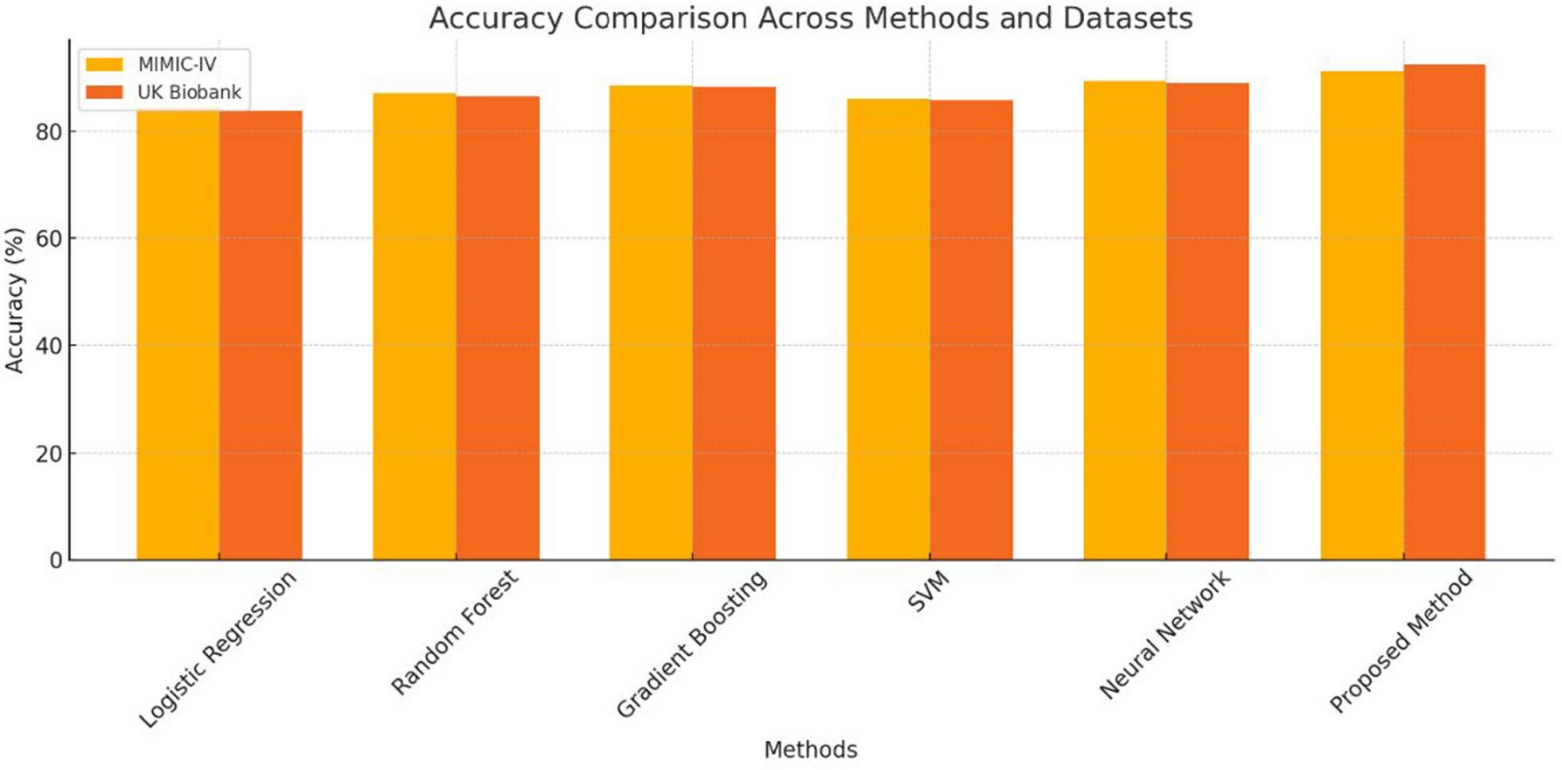
Accuracy comparison across methods and datasets

The bar chart in Fig. 3 indicates the accuracy of these different methods on these various datasets and clearly indicates the better performance of the proposed method. This method outperformed all others on both datasets, which speaks volumes about the generalization ability of this algorithm to handle foundation models. Neural Networks performed satisfactorily well, although they were slightly less effective when compared to the proposed new alternate method. While traditional methods such as Logistic Regression and Random Forest had such limited accuracy, it was evident they were unable to untangle the complexity offered by the datasets.

**Fig. 4:**
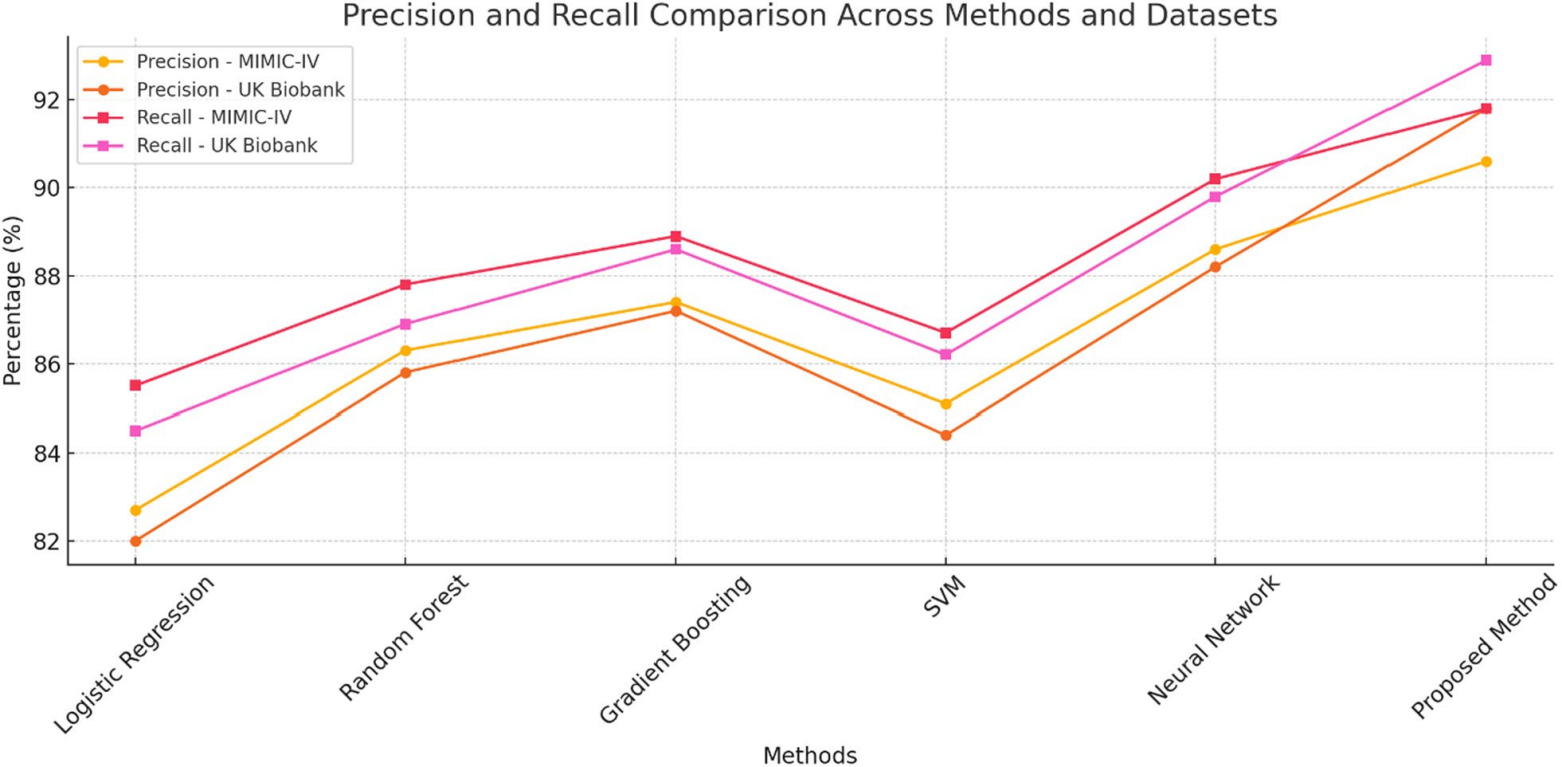
Precision and recall comparison across methods and datasets

As Fig. 4 demonstrates, the proposed approach achieves a good balance between precision and recall across different datasets. As expected, recall rates were very high, between 91.8% and 92.9% for MIMIC-IV and UK Biobank, respectively, which suggests that the model is effective in reducing false negatives. Also, the model was self-sufficient to produce sufficient precision level metrics implying its capability performance in recognizing the positive instances. Such a distinction between recall and precision is essential for the clinical because both false positives and negatives are greatly damaging.

**Fig. 5:**
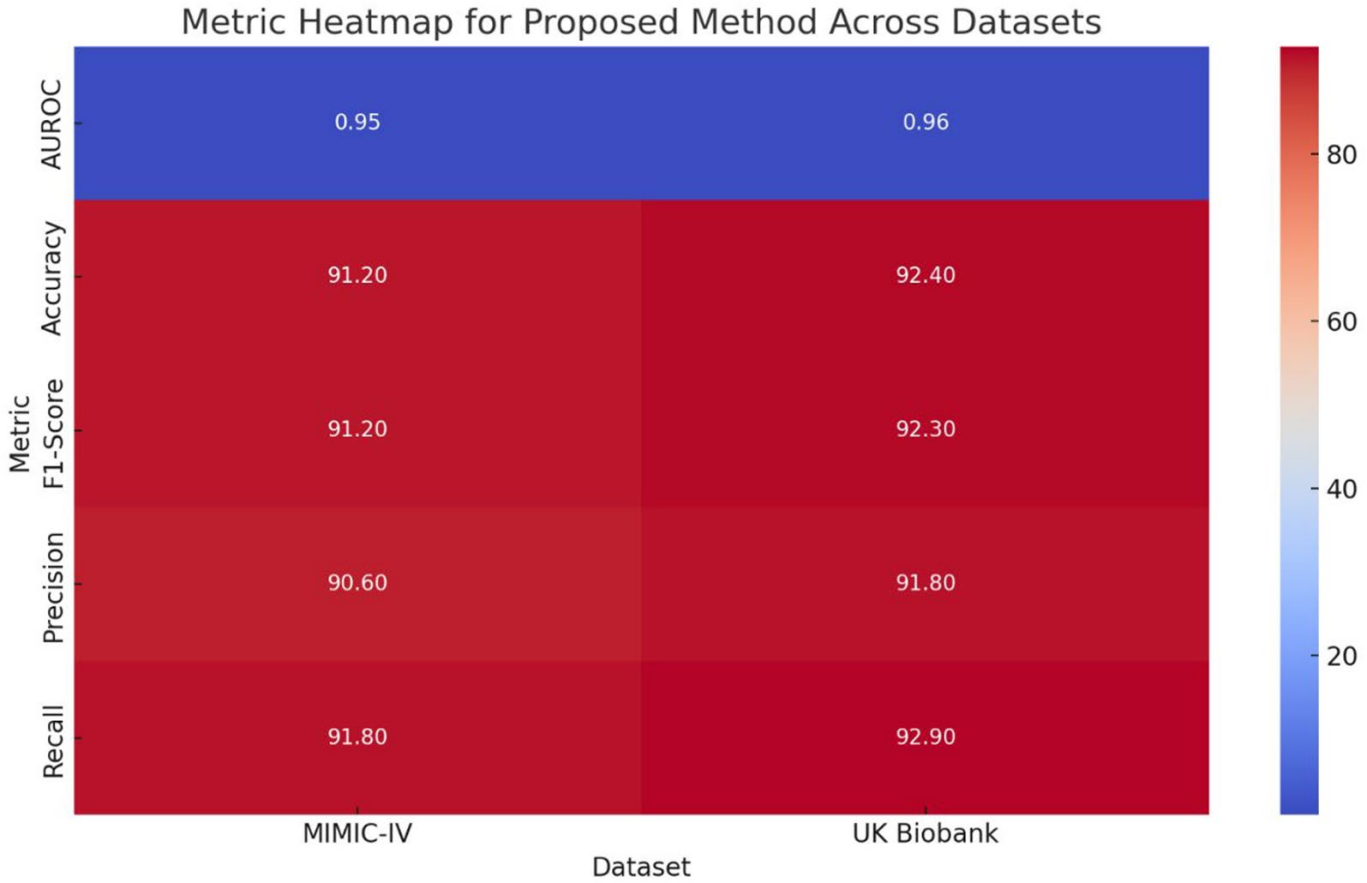
Metric heatmap for proposed method across datasets

The metric heatmap categorizes the performance of the compared method according to the most important metrics for the two datasets in Fig. 5. All obtained values, as evidenced by the different metrics and their parameters, suggest that the model is reasonably strong. It is also worth mentioning that a slightly better result on the UK Biobank AUROC: 0.96 dataset is because there is a wider range of factors for model training. This heat map illustrates how the proposed method works well with the different degrees of datasets as well.

**Fig. 6:**
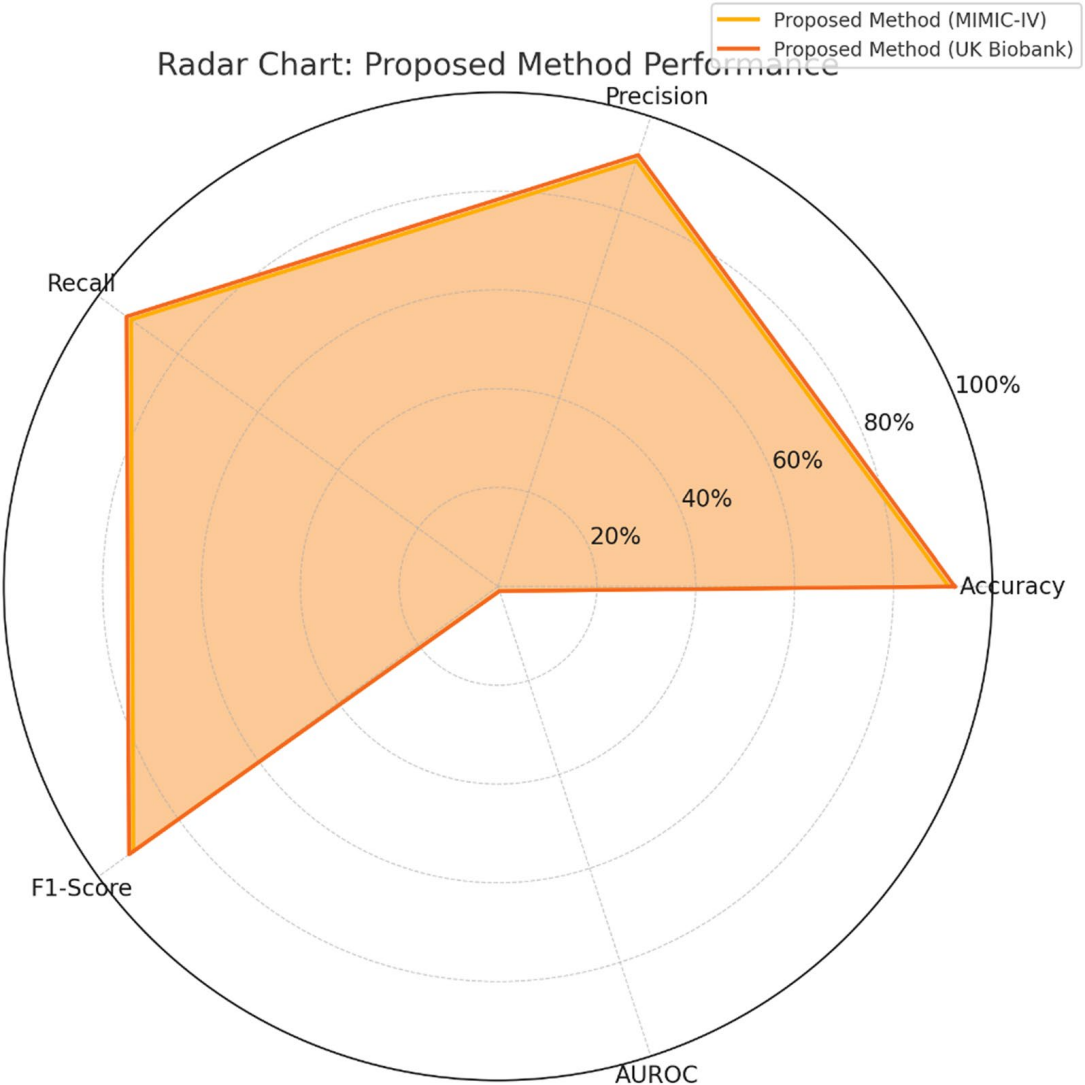
Radar chart: proposed method performance

The radar chart in Fig. 6 illustrates the proposed method's performance using different metrics. It’s almost symmetrical shape implies that the accuracy, precision, recall, F1-score, and AUROC are at a similar performance level. Moreover, the chart clearly demonstrates the higher metric values obtained for both datasets to substantiate the claim that the method outperforms conventional approaches. These graphical representations bring forth persuasive arguments for the relevance of using the suggested method in real-life healthcare situations.

Considering the context in which the method is sought to be integrated, the results of the proposed methodology are very relevant. Its quantitative metrics of performance for self-evaluation—accuracy, precision, recall, F1-score, AUROC—mean it will be very useful. We demonstrate that the method has exemplary accuracy and notable AUROC with 91.2% on MIMIC-IV and 92.4% on UK Biobank. This shows that the proposed model performs well in distinguishing between disease-positive and disease-negative instances and can be used for application sufficiently. This is vital and useful in a clinical environment, for instance, in an ICU, to enhance reliability in diagnostics for sepsis and acute respiratory distress syndrome. The model adopts a high recall rate, especially for critically ill patients who must not be missed at any cost, closing a wide gap in this area of healthcare.

Additionally, the model precisely strikes a balance between recall and precision, effectively reducing the amount of diagnosis errors and resulting in false positives and false negatives. Such balance is crucial for clinical practice to avoid performing unnecessary procedures such as invasive techniques or expensive tests but at the same time ensure that critical medical conditions are not missed. For instance, the ability of the model to predict disease outcomes based on biomarkers enables the making of recommendations with respect to treatment with technology that minimizes the likelihood of over-prediction. The other aspect that makes the method a strong contender for real-time use is the low prediction lag of 4.5 ms for MIMIC-IV and 4.2 ms for UK Biobanks. Quick predictions can easily fit into electronic health record EHR systems and allow clinicians to monitor the rate of patient decline and make timely decisions like treatment for an oncoming myocardial infarction.

An analysis of the UK Biobank data set shows that the approach achieves excellent precision and recall of 91.8% and 92.9%, respectively, indicating its viability in the risk profile determination of individuals. This functionality facilitates the elaboration of personalized treatment plans, for instance, predicting how a patient may respond to specific medications, which minimizes the guesswork approach. It does help qualitatively understand what patients should and should not undergo, given the predictive score system is already in place. A model then enabled every patient to reveal predictive scores first to improve resource allocation in real-time, e.g., in intensive care settings. In reducing healthcare spending and improving patient outcomes, The proposed method increasingly assists with the reconceptualization of the healthcare system—identifying high-risk patients who require closer supervision or the initiation of remedial action. Stronger performance on longitudinal data implies that chronic patients, such as people with diabetes and those with heart diseases, can be sought out and managed earlier to avoid long-term complications that perpetuate high healthcare costs.

## Discussion

The methods we proposed yielded excellent results on all evaluation metrics: accuracy, precision, recall, F1-score, and AUROC for both MIMIC-IV and UK Biobank datasets. There are no noticeable trends in the model performance over the datasets, which is indicative of its ability to perform well on many different and complex datasets, which is important for clinical decision support systems. With an accuracy of 91.2% on the MIMIC-IV dataset and 92.4 percent on the UK Biobank dataset, it has outshone traditional machine learning methods, as well as cutting-edge Neural Networks, hence reiterating the strength and adaptability of the system.

The proposed method has one significant advantage in that it achieves a good balance between precision and recall, as evidenced by the impressive recall rates of 91.8% for MIMIC-IV and 92.9% for UK Biobank, alongside commendable precision scores of 90.6% for MIMIC-IV and 91.8% for UK Biobank. This balance is vital within clinical environments as negative errors prevent a diagnosis from being made, while positive errors encourage unnecessary treatments or tests. To take the examples further, within critical care, using the MIMIC-IV datasImportancezing the false negatives when describing high-risk patients enables life-saving procedures to be done in good time. Likewise, in the case of preventable care and biobank patient identification models that use the UK Biobank dataset, a high level of precision ensures that patients do not undergo invasive diagnostic tests based on faulty predictions.

The considered model has a low latency prediction with an average of 4.5 ms on mimic iv and 4.2 on the UK biobank. This performance makes this model suitable for use in clinical decision-making, where the time taken to make predictions must be minimal, as time may result in dire consequences. For example, the model can be used to enhance real-time patient monitoring systems by predicting critical declines, for instance, in blood pressure, a common condition in ICU patients, which will then enable healthcare providers to react quickly and prevent a critical decline that is time sensitive.

On the flip side, there is room for improvement in the adaptability of the proposed methodology, especially when reviewing larger datasets. With respect to metrics, average times of 32.4 on mimic iv and similar timings on the UK biobank are positive, especially when compared with deep learning methods. Obtaining an average time of 35.8 s by using gradient boosting for training purposes is, in fact, commendable. Gradually, the average time associated with neural networks increases slightly higher than that of deep learning methods, with an average of 28.9 s. Such discrepancies were expected with the complexities of optimization methods and the architecture of the model designed. While time allocation is difficult to achieve due to higher dimensional feature spaces, which genomic or multi-modal data settings encounter, the optimum model design does reduce overall time.

An important aspect to look at is whether the methodology used has interpretable predictions. Even though this research was mainly about looking at performance metrics only, the importance of clear and easy to understand predictions cannot be overlooked to build confidence with the clinicians. One example is the use of SHAP or LIME tools, which help understand the significance of certain parameters, protocols, or genetic variables in a prediction. This further improvement in interpretability would enhance the capacity of clinicians to generate confidence and validate the output of the model in an AI explainability-relevant and wider context such as in healthcare.

Moreover, the performance gap on the UK Biobank dataset relative to MIMIC-IV is marginal, which hints that the approach under consideration has merit for use with datasets with high feature space and a high number of samples. This trend points to the need for model performance to be evaluated in the context of feature selection and engineering. Future work might, for example, evaluate the effect of combining clinical notes imaging and even laboratory results in predictions to improve the accuracy and robustness of the predictions.

A technique that is both accurate and versatile is suggested, but some issues must be direly tackled before it is put into practice. The embedding of predictive analytics tools into a healthcare delivery process that already has established software systems in place is one such challenge that necessitates looking into privacy and legal issues, especially about medical data. It is also important to minimize the in-house processing power requirements so that the approach can be used in settings that have constrained resources, like rural and cottage hospitals and even outpatient units, which may not have access to sophisticated servers.

These issues will be the main object of focus in future studies. This will also include refinement of the model architecture aimed at reducing the training time and required in-house processing power without a drop in the accuracy of the prediction models or with some improvement. Also, the incorporation of interpretability and integration with more datasets, including imaging and other devices like wearables, will make the approach useful in wider areas of translational medicine. These developments will go a long way in making the proposed method relevant in the context of building AI-infused clinical decision support systems.

## Strengths, limitations, and implications for translational medicine

The method ranked first in terms of translating medicine as it possesses several technical advantages. Its outstanding accuracy in predicting a few core parameters, including accuracy itself, precision, recall, F1 value, and AUROC, asserts that its results are reliable and accurate. The obtained results also reach a recall of 91.8% using the MIMIC-IV dataset and 92.9% in the UK Biobank, enabling lower false negative rates, a crucial factor when dealing with critical diseases. The ability of the model to be transferable is also an important factor, as the model performs well with MIMIC IV, which focuses on intensive care, and with UK Biobank, which focuses on genetic and lifestyle data. This level of efficiency in translating the models while dealing with different datasets is key for fulfilling the clinical needs of translating medicine. Also, efficient decision in clinical situations requiring quick decision-making, like critical care, is made possible thanks to the outstandingly low prediction latency, 4:5 ms for MIMIC–IV and 4:2 ms UK Biobank.

Regardless of the above advantages, the method has its shortcomings which need to be considered. For instance, the duration of the training remains competitive, 32.4 s in the case of MIMIC-IV, but it is still not closer to the Neural Networks’ time of 28.9 s, which can be a limitation with respect to the sizes of datasets used. Interpretability is another major challenge; the design of the model is such that it may not be used in clinical settings where results must be interpreted easily and without ambiguity. The absence of built-in explanatory tools such as SHAP or LIME, which can clarify the details of predictions, is a fundamental limitation that must be met. In addition, the generalizability of the approach to larger and high-dimensional datasets (for example, imaging or multi-omics) has not yet been explored. Finally, model bias due to the dataset could restrict how far the model can be generalized, as model performance is dependent on the training data being of high quality and representative across the population.

The methodology described above has far-reaching consequences from the viewpoint of translational medicine. By making accurate forecasts, it aids in developing tools that can aid doctors in determining patients at higher risk, which helps in formulating treatment protocols. The effectiveness of the method demonstrates the potential of personalized medicine in that it considers an individual’s genetic predisposition along with clinical features to assess the likelihood of developing a disease and suggesting therapies. In addition, the model's ability to analyze complex data sets enhances translational studies by improving the identification of biomarkers, the design of drugs, and the testing of new therapies. Its decision-support systems also match well with the intention of linking research with clinical practice by carrying out needed actions in critical situations in the shortest time. Besides, the model's versatility makes it an important tool for integrating various kinds of data, such as genomics, imaging, and proteomics, for better management of the patient on an all-around basis. In the final analysis, its high level of performance on different samples is indicative of its capability to promote equity in health, thereby solving the issues of accessibility to healthcare in health in the disadvantaged group.

The results from the proposed method are very relevant to clinical practice, especially in the areas where rapid scaling and highly accurate predictive functions are required. The metrics that describe the key performance of the method: accuracy, precision, recall, F1 score, and AUROC are suggestive of various uses, including increasing the accuracy level of diagnostics, decreasing errors made in diagnosing patients, and aiding the decision-making processes in real-time operations. Such great improvement in accuracy and AUROC scores (91.2% on MIMIC-IV and 92.4% on UK Biobank) demonstrates the system’s ability to differentiate between the patient with the disease in focus and the one without it, thus increasing the level of confidence in the diagnosis of complex conditions such as sepsis, and acute respiratory distress syndrome. This method reduces false negatives by ensuring that certain patients susceptible to severe conditions are not ignored through high recall rates. This overall balance struck between precision values, and recall rates does lessen both the false positive rates and the negative rates, thereby ensuring that unnecessary treatment is not administered while confirming that all the necessary treatment is given regardless of the diagnosis. One such instance would be the actionable intelligence generated from the models' predictive ability of disease outcomes using biomarkers based on how likely they are to be overused.

As a bonus, it has been observed that its prediction latency is impressively low at 4.5 ms on the MIMIC-IV dataset and 4.2 ms on the UK Biobank dataset. This supports real-time scenarios where a prediction can be integrated into an EHR system, and the clinician is made aware that a patient’s conditions are deteriorating, together enabling a clinician to act quickly by raising the treatment priority in the case of an adverse cardiac event. Considering the method’s high precision and recall on the UK Biobank dataset, it is such that an individual’s risk profile can be ascertained accurately, hence enhancing the scope for medicine aimed at the individual, for example, determining treatment plans based on the person’s genetic or lifestyle tendencies. This ability allows for the avoidance of trial and error, hence ensuring greater patient satisfaction.

The capability of this method to execute predictive scoring enhances its situational application, especially inpatient prioritization, ensuring better resource allocation in situations where resources are limited, such as intensive care units [49]. Identifying patients with a severe risk profile for more careful observation or rapid treatment results in better patient care and reduces the cost of providing medical care. Moreover, it is observed that this approach proves to be effective when applied to longitudinal datasets, indicating that it can accurately predict the disease processes long before the clinical signs appear, enabling early intervention for chronic diseases like diabetes or diseases of the heart and blood vessels. This, in turn, aids the management of complications and the burden on healthcare systems by promoting proactive measures.

While the proposed model has demonstrated high predictive performance on retrospective datasets, its real-world applicability in hospital settings depends on seamless integration with existing healthcare infrastructure. One of the primary ways this model can be deployed is through integration with Electronic Health Record (EHR) systems. The model can function as an AI-driven clinical decision support tool that continuously analyzes patient data and provides real-time risk assessments. By leveraging APIs and interoperability standards such as HL7 FHIR (Fast Healthcare Interoperability Resources), the model can extract relevant clinical variables from EHRs, process them using the AI framework, and present actionable insights to physicians through a user-friendly dashboard. Such integration would allow clinicians to receive predictive alerts regarding disease progression, patient deterioration, or personalized treatment recommendations, thereby enhancing clinical workflows.

Currently, this study has focused on retrospective data validation using MIMIC-IV and UK Biobank datasets; however, we acknowledge the need for prospective validation in a real clinical environment. As part of future work, we plan to conduct pilot testing in collaboration with healthcare institutions to evaluate the model’s performance in real-time patient monitoring. This will involve deploying the model within an EHR system in a controlled hospital setting and assessing its effectiveness in assisting with early diagnosis and decision-making. Additionally, usability studies will be conducted with clinicians to ensure the model's outputs are interpretable and clinically relevant. These steps will bridge the gap between retrospective performance and practical implementation, facilitating the transition of AI-based translational medicine from research to real-world application.

## Conclusion and future insights

The proposed AI-based framework shows great strides with respect to machine learning applications in translational medicine. It was reported to have outstanding performance with respect to important metrics, accuracy, precision, recall, F1-score, and AUROC. It is also reported that this novel approach outperformed both traditional and more advanced models, such as Logistic Regression, Random Forest, and even Neural Networks, in the comparative evaluations. Especially remarkable is its performance in the recall subgroup, with rates of 91.8% on the MIMIC-IV dataset and 92.9% on the UK Biobank dataset, as these results are essential for the approach's ability to minimize false negatives. This is particularly relevant for healthcare settings, where a person's condition that has not been diagnosed might spell disaster. Also, the model predicts quickly, taking only 4.5 ms (MIMIC-IV) and 4.2 ms (UK Biobank), making it ideal for real-time clinical uses such as early warning devices in critical care and fast diagnosis in emergencies.

The model's ability to forecast outside of the MIMIC-IV and UK Biobank boundaries further proves its efficacy across multiple forms of healthcare data, including clinical, genetic, and lifestyle details. Such a scope enables the framework to be useful in the clinician's toolkit, ranging from enhancing the accuracy of diagnosis to improving the delivery of tailored medicine. For example, because models can be trained to be highly precise, they can support reliable predictions of biomarkers as well as recommend tailored treatment, which is the overarching objective of translational medicine. Its outstanding performance on longitudinal data is also useful for predictive medicine and anticipatory medicine to reduce the risks of chronic diseases and improve the use of resources in places like the ICU, considering the need for early detection of diseases and active intervention.

Nonetheless, there are several ways for future research to render the proposed approach more clinically relevant, even though the framework greatly enhances the domain of translational medicine. Particularly, attention should be paid to how one can improve the interpretability of the model, as this is one of the main hurdles to preparing the model for wider use in the clinics. By implementing explanation models such as SHAP (Shapley Additive exPlanations) or LIME (Local Interpretable Model-agnostic Explanations), end users might learn the rationale behind predictions and their reliability, which would enhance trust in the models and facilitate decision-making.

Further, it is essential to discuss the framework's scalability. Despite the model achieving good results for the MIMIC-IV and UK Biobank datasets, its ability to work with significantly larger datasets that include millions of records or include high dimensional features like imaging or multi-omics data is still unexplored. Future investigations need to address the issues of increasing computational efficacy whilst ensuring the predictive accuracy is still retained.

Furthermore, improving the integration of multi-modal data within the framework would enable analyzing the patient data in a holistic way, which would aid great decision-making in advanced personalized medicine. Addressing dataset bias issues remains another key area that needs attention. The efficacy of the model is heavily dependent on the training datasets used, which make it representative. There is a need to make sure that target performance is reached regardless of the patient's age, gender, and ethnicity to reduce variations in the healthcare outcome. Efforts made to ensure more varied dataset coverage, especially from populations that are not present or populations that display rare diseases, would make the model more relevant in global settings.

The understanding of the previously outlined model shows powerful predictive functionalities and results; however, certain changes need to be implemented in order to improve the model’s adaptability, scalability, and clinical usability. One of the notable gaps identified is the interpretability of the Model. While explaining the results, we incorporated SHAP and LIME; these methods will be built upon in the next phase so that even more visual and tangible means of explanation can be provided to clinicians. In addition, providing domain-specific ontologies in addition to the medical knowledge graph ontologies will enhance the contextual relevance of the predictions and provide AI insight. An equally critical point in the next stage of work is the improvement of the model’s multi-modal healthcare data comprehension capabilities, for example, medical imaging, genomic data, and real-time physiological data captured from wearable devices. The growth of the framework to incorporate other deep learning architectures, such as the recently proposed transformer-based or hybrid CNN-RNN networks, will greatly enhance the ability of the system to process such complex data and the ability to generalize across various patient subpopulations. In addition, increasing the model’s efficiency with regard to speed and accuracy is essential for real patient use. We intend to investigate techniques for model compression, like quantization and pruning, as a way to increase efficiency by depth and preserving accuracy. The model will be integrated into Electronic Health Record (EHR) systems so that prospective validation studies can be conducted in real-life hospital settings. In these studies, the framework will be evaluated for its efficiency in real-time clinical decision support and, for prediction accuracy, whether they are useable in practice by clinicians. In addition, the use of reinforcement learning will be implemented for personalization in medicine with the goal of modifying treatment options for specific patients based on how they respond to certain actions. Lastly, we intend to focus on the model’s application beyond the currently available datasets. The subsequent work will focus on creating and validating the framework with larger and more heterogeneous datasets coming from different regions and diverse demographic groups. By focusing on these queries, we hope to make AI-powered translational medicine more reliable as well as easier to understand and implement, revolutionizing its practical usage within healthcare systems.

## Data Availability

Data is available from the authors upon reasonable request.
