# Correction: Artificial intelligence-driven translational medicine: a machine learning framework for predicting disease outcomes and optimizing patient-centric care

**DOI:** 10.1186/s12967-025-06948-8

**Published:** 2025-08-14

**Authors:** Laith Abualigah, Saleh Ali Alomari, Mohammad H. Almomani, Raed Abu Zitar, Kashif Saleem, Hazem Migdady, Vaclav Snasel, Aseel Smerat, Absalom E. Ezugwu

**Affiliations:** 1 Computer Science Department, Al Al-Bayt University, Mafraq, 25113 Jordan; 2 Faculty of Science and Information Technology, Jadara University, Irbid, 21110 Jordan; 3 Department of Mathematics, Facility of Science, The Hashemite University, P.O box 330127, Zarqa, 13133 Jordan; 4 Faculty of Engineering and Computing, Liwa College, Abu Dhabi, United Arab Emirates; 5 Department of Computer Science and Engineering, College of Applied Studies and Community Service, King Saud University, 11362 Riyadh, Saudi Arabia; 6 CSMIS Department, Oman College of Management and Technology, 320 Barka, Oman; 7 Faculty of Electrical Engineering and Computer Science, VŠB-Technical University of Ostrava, 70800 Poruba‑Ostrava, Czech Republic; 8 Faculty of Educational Sciences, Al-Ahliyya Amman University, Amman, 19328 Jordan; 9 Centre for Research Impact and Outcome, Chitkara University Institute of Engineering and Technology, Chitkara University, Rajpura, Punjab 140401 India; 10 Unit for Data Science and Computing, North-West University, 11 Hofman Street, Potchefstroom, 2520 South Africa

**Correction to: Journal of Translational Medicine (2025) 23:302** 10.1186/s12967-025-06308-6

Following publication of the original article [1], it was identified that in the paragraph beginning “An analysis of the UK Biobank data…” the phrase “Lloyds increasingly assists with the reconceptualization of the healthcare system…” should have read “The proposed method increasingly assists with the reconceptualization of the healthcare system…”.

Additionally, in the paragraph beginning “The obtained results also reach a recall…” the values “91:8%” and “94:9” should have read “91.8%” and “92.9%”.

The original article [1] has been corrected.
